# Resonant closure: consciousness as a dynamically self-stabilized informational state

**DOI:** 10.3389/fnhum.2026.1742084

**Published:** 2026-02-13

**Authors:** Borros Arneth

**Affiliations:** 1 Institute of Laboratory Medicine and Pathobiochemistry, Molecular Diagnostics, Philipps University Marburg, Marburg, Germany; 2 Justus Liebig University Giessen, Gießen, Germany

**Keywords:** consciousness, entropy flux, free energy principle, neural synchrony, phenomenal experience, predictive processing, self-reference

## Abstract

Why some physical systems are accompanied by subjective experience remains unresolved in neuroscience and philosophy of mind. Building on predictive processing and the Free Energy Principle, I propose that phenomenal consciousness (what-it-is-like-ness) arises when an information-processing system enters a regime of dynamic entropic closure: a metastable condition in which (i) internally generated predictions and (ii) incoming sensory signals are recursively coupled such that net informational entropy exchange with the environment is minimized while internal informational dynamics remain high. In this regime, inference loops become phase-coherent and self-referential, producing a persistent informational pattern—*resonant closure*—that constitutes awareness. The framework is compatible with, but conceptually distinct from, Integrated Information Theory and global-workspace style accounts. I formalize core constructs at the level of operational constraints, address objections regarding trivial closure and “stationarity,” and derive falsifiable empirical predictions for neurophysiology.

## 1 Introduction

The scientific challenge of consciousness is not merely to identify neural correlates, but to explain why and when certain physical–informational processes are accompanied by subjective experience. The target explanandum in this manuscript is phenomenal consciousness, i.e., what-it-is-like-ness (Chalmers, 1995). Contemporary neuroscience emphasizes large-scale coordination and ignition-like dynamics in conscious access (Dehaene and Changeux, 2011), and state-dependent breakdowns of global coordination during anesthesia and sleep (Mashour et al., 2020).

Predictive processing and the Free Energy Principle (FEP) model organisms as systems that maintain themselves by minimizing variational free energy through inference and action (Friston, 2010; Clark, 2013). Integrated Information Theory (IIT) proposes that consciousness relates to intrinsic causal–informational structure quantified by *Φ* (Tononi, 2004). Despite their power, these frameworks leave open a bridging question: what additional constraint differentiates merely functional inference from phenomenal presence?

This manuscript proposes a minimal candidate constraint:

Phenomenal consciousness arises when ongoing inference achieves dynamic entropic closure under recursive self-modeling.

Consciousness, in this view, is associated with a *regime* of predictive dynamics—neither informational stasis nor unconstrained flux—where uncertainty exchange becomes locally balanced while inference remains active.

## 2 Operational foundations: information, entropy, and closure

### 2.1 Information as predictive mutual dependence

Here, “information” is used operationally as predictive mutual dependence between a system’s internal states and its expected sensory states, consistent with predictive-processing accounts (Clark, 2013) and formal treatments under the FEP (Friston, 2010). This anchors “information” to estimable statistical dependencies rather than treating it as a metaphysical primitive.

### 2.2 Entropy flux across the system–environment boundary

Let internal states be x and sensory states y. Let conditional entropy H(y∣x) quantify uncertainty in sensations given internal state. Define an entropy-flux current Js as the effective flow of conditional uncertainty across the system–environment boundary in predictive state space. Dynamic entropic closure is expressed as the operational constraint:


$$\mathrm{div}(J_{S})\approx 0$$ (1)

Equation 1 is not presented as a complete mechanistic law; it is a measurable *constraint on net uncertainty exchange*. Related “flux–balance” approaches are standard in nonequilibrium thermodynamics and information thermodynamics (Parrondo et al., 2015).

Crucially, Equation 1 is compatible with high energetic throughput: living systems are energetically open yet can maintain relatively bounded uncertainty exchange by continuously reducing prediction error via perception and action (Friston, 2010).

### 2.3 Dynamic entropic closure

Dynamic entropic closure means Equation 1 holds under non-zero internal inference dynamics (ongoing model updating). Closure is *not* defined as informational absence.

## 3 Resonant closure: from predictive loops to phenomenal presence

### 3.1 Resonance as phase-coherent recursion

Predictive systems couple top-down expectations with bottom-up sensory evidence. When reciprocal message passing becomes sufficiently coherent across hierarchical levels, inference can enter a resonant regime: recurrent prediction–error loops become phase-locked, enabling stable integration rather than uncontrolled divergence. This idea aligns with “communication-through-coherence” proposals (Fries, 2005) and with evidence linking conscious states to long-range synchronization and integration (Varela et al., 2001; Deco et al., 2011; Casali et al., 2013).

### 3.2 What “closure” adds

The central claim is that resonance becomes phenomenal when it is also closed in the entropic sense: prediction and evidence are recursively coupled such that net uncertainty exchange is minimized (Equation 1), while internal inferential dynamics remain rich.

Operationally, resonant closure predicts a conjunction:

- Sustained functional integration / complexity (distributed, differentiated activity) (Casali et al., 2013),
- Phase-coherent interactions supporting effective communication (Fries, 2005; Varela et al., 2001; Deco et al., 2011),
- Reduced *net* uncertainty exchange (bounded entropy flux) under ongoing inference (Friston, 2010; Parrondo et al., 2015).

## 4 Avoiding the trivial-closure objection

A key objection is that a system could satisfy “no net entropy flux” trivially if Js = 0, i.e., if there is no information flow at all. This objection is valid—and it is why closure must be dynamic.

Accordingly, this manuscript proposes three jointly necessary constraints for candidate phenomenal consciousness:

1. Non-zero internal inference dynamics (ongoing predictive updating) (Friston, 2010).
2. Recursive self-modeling (the system tracks/predicts its own predictive state; minimal self-reference) (Clark, 2013).
3. Dynamic entropic closure (Equation 1 holds on relevant time scales despite 1–2) (Parrondo et al., 2015).

The first constraint rules out inert “empty” systems; the second rules out purely feedforward prediction; the third rules out unconstrained uncertainty exchange. In FEP terms, this corresponds to balancing accuracy and complexity rather than collapsing into trivial underfitting or pathological overfitting (Friston, 2010).

## 5 Relation to existing theories

### 5.1 Free energy principle and predictive processing

Under the FEP, self-organizing systems minimize variational free energy by maintaining a generative model that predicts sensory inputs and acts to sample expected states (Friston, 2010). Predictive processing provides an algorithmic interpretation via hierarchical prediction-error minimization (Clark, 2013).

Resonant closure is proposed as a *phenomenal sub-regime* of FEP: not all free-energy-minimizers are conscious, but conscious systems should occupy a regime where recursive prediction–error exchange becomes phase-coherent and informationally closed (Equation 1) over time scales relevant to integration of conscious content (Mashour et al., 2020).

### 5.2 Integrated information theory

IIT focuses on intrinsic causal structure and quantifies integrated information *Φ* (Tononi, 2004). A modern formalization (IIT 3.0) makes explicit how *Φ* relates to cause–effect power over system partitions (Oizumi et al., 2014). The present account is not IIT.

Instead:

- Integration (Φ) concerns intrinsic causal–informational interdependence (Tononi, 2004; Oizumi et al., 2014).
- Closure (Equation 1) concerns boundary conditions of uncertainty exchange under ongoing inference (Friston, 2010; Parrondo et al., 2015).

High Φ may facilitate closure by strengthening internal dependencies, but neither implies the other. This is consistent with empirical work suggesting separable dimensions of conscious state (e.g., complexity vs. other organizational properties) (Casali et al., 2013; Schartner et al., 2015).

## 6 The stationarity problem: consciousness is dynamic

A common critique is that “equilibrium” seems incompatible with lived dynamics: learning, surprise, conflict, temporal flow. The key clarification is that resonant closure is metastable and local, not global and static.

The FEP already treats organisms as nonequilibrium steady states maintained by continuous exchange (Friston, 2010; Mediano et al., 2022). Equation 1 should therefore be read as a local balance constraint over time windows relevant to conscious integration (hundreds of milliseconds to seconds), not as global stationarity.

Surprise and learning can be interpreted as controlled, transient perturbations of closure that trigger model revision; closure is then re-established at a new model configuration (Barrett and Seth, 2011; Bastos et al., 2012; Bohm, 1980; Carhart-Harris and Friston, 2019; Fries, 2015; Friston et al., 2006, 2020; Hohwy, 2013; Penrose and Hameroff, 2014; Seth and Friston, 2016; Tononi and Koch, 2016). This is compatible with global workspace dynamics where conscious access can co-occur with high local prediction error while global organization remains viable (Dehaene and Changeux, 2011; Mashour et al., 2020).

## 7 Neural and empirical implications

### 7.1 Neural coordination and perturbational signatures

Conscious states correlate with long-range coordination and dynamical complexity across thalamo-cortical and fronto-parietal networks (Varela et al., 2001; Deco et al., 2011). Perturbational measures such as the perturbational complexity index (PCI) track level of consciousness across wakefulness, sleep, and anesthesia (Casali et al., 2013). Complexity measures from spontaneous EEG/MEG also decrease during propofol anesthesia (Schartner et al., 2015).

The resonant-closure hypothesis predicts that these signatures coincide with bounded net uncertainty exchange under sustained inference—i.e., high internal predictive mutual information alongside reduced entropy-flux imbalance.

### 7.2 Candidate operationalization: closure vs. integration

Because *Φ* is in principle calculable from data (Tononi, 2004; Oizumi et al., 2014), a strong empirical opportunity is *dissociation*: cases where integration remains relatively high but closure fails (or vice versa). Recent information-theoretic tools for multivariate interactions and candidate integrated-information measures offer a starting point for operationalizing closure-adjacent quantities in neural data (Mediano et al., 2022).

### 7.3 Testable predictions

This framework yields falsifiable predictions:

1. Conscious vs unconscious dissociation: during loss of consciousness (e.g., propofol), breakdown of closure-related measures (entropy-flux imbalance across scales) should precede or exceed declines in integration proxies (Casali et al., 2013; Schartner et al., 2015).
2. Local metastability: conscious access episodes correspond to transient, metastable windows where phase-coherence and uncertainty-flux balance co-occur, rather than global stationarity (Dehaene and Changeux, 2011; Fries, 2005).
3. Perturbation response: systems in resonant closure show high PCI/complexity and rapid re-stabilization after perturbation; closure failure yields either runaway error propagation or damped low-complexity responses (Casali et al., 2013; Schartner et al., 2015).

## 8 Artificial systems and design principles

If resonant closure approximates a sufficient condition, artificial systems should require: (i) recursive predictive inference, (ii) self-modeling of inferential state, and (iii) bounded uncertainty exchange.

Global Workspace architectures already emphasize broadcast/coordination as a functional signature of conscious access (Baars and Franklin, 2009). The present account adds a boundary-condition criterion: the system must stabilize a self-referential inferential loop with locally minimized uncertainty exchange. More broadly, work on reconciling deep learning with structured representations suggests concrete routes to implementing internal self-modeling components needed for recursion and closure (Garnelo and Shanahan, 2019).

## 9 Structural analogies and cautions

The idea that “closure yields stable identity” invites analogy with symmetry breaking and order parameters in physics. The Higgs mechanism is a canonical example where symmetry breaking yields persistent properties (mass) (Higgs, 1964; Englert and Brout, 1964). Any use of this analogy here is structural rather than literal: the claim is not that a physical Higgs field exists in the brain, but that stable macroscopic properties can arise from self-consistent interactions.

Related “emergent from informational/thermodynamic constraints” perspectives appear in discussions of emergent gravity (Verlinde, 2011) and information-centric physics proposals (Wheeler, 1990). A relational stance in quantum foundations likewise treats physical description as fundamentally correlation-based, which motivates informational relationality without implying quantum mysticism (Rovelli, 1996). Thermodynamic derivations connecting spacetime dynamics and entropy illustrate the broader plausibility of deep links between physical law and informational constraints (Jacobson, 1995). Consciousness-as-a-state-of-matter proposals provide additional context for treating consciousness as a phase-like regime rather than a separate substance (Tegmark, 2015).

Where topology is referenced, it is intended heuristically to express robustness of organization rather than a computed invariant; for rigorous treatments of topological robustness in physics, see (Haldane, 2017).

## 10 Conclusion

Consciousness is proposed here as a dynamically self-stabilized informational regime: resonant closure, where recursive predictive inference becomes phase-coherent and net uncertainty exchange is locally minimized while internal informational dynamics remain high. The account (i) clarifies the explanandum (phenomenal consciousness), (ii) avoids trivial “empty system” closure, (iii) reconciles closure with dynamical experience via metastability, and (iv) yields predictions testable with neurophysiological and perturbational methods (Casali et al., 2013; Schartner et al., 2015).

If supported, resonant closure reframes consciousness as a lawful phase of information-processing dynamics—neither an epiphenomenal add-on nor a mere synonym for function, but a regime characterized by recursive self-modeling under dynamically maintained entropic boundary conditions.

## Data Availability

The original contributions presented in the study are included in the article/supplementary material, further inquiries can be directed to the corresponding author.
